# Synthesis and structure of 4-[(2,3,4,5,6-pentafluoro­phen­oxy)carbon­yl]phenyl 4-(tetra­dec­yloxy)benzoate

**DOI:** 10.1107/S2056989026006213

**Published:** 2026-06-23

**Authors:** Khaleel Ahmed, H. C. Devarajegowda, B. Bommalingaiah, G. N. Venkatareddy, H. T. Srinivasa, B. S. Palakshamurthy

**Affiliations:** a Department of Physics Yuvaraja's College University of Mysore,Mysore-570005 Karnaataka India; b Department of Physics, Government Science College, Chithradurga-577501, Kanataka, India; c Raman Research Institute, C V Raman Avenue Sadashivanagar Bangalore-560080 Karnataka India; d Department of PG Studies and Research in Physics Albert Einstein Block UCS Tumkur University, Tumkur Karnataka-572103 India; University of Aberdeen, United Kingdom

**Keywords:** crystal structure, tetra­dec­yloxy, penta­fluoro­phen­oxy, Hirshfeld surface, hydrogen bond

## Abstract

In the title mol­ecule, C_34_H_37_F_5_O_5_, the dihedral angles between the central carbonyl­phenyl and adjacent perfluoro­phen­oxy and (tetra­dec­yloxy)benzoate rings are 74.19 (2) and 67.86 (2)°, respectively and the tetra­decyl chain adopts an extended conformation. In the crystal, the mol­ecules are linked by C—H⋯O and C—H⋯F hydrogen bonds, forming *C*(7) and *C*(10) chains, respectively, both running infinitely along [010]. The Hirshfeld surface analysis reveals that the major contributions to the two dimensional fingerprint plots are from H⋯H (49.4%), F⋯H/H⋯F (16.7%) and O⋯H/H⋯O (9.0%) contacts. An inter­molecular inter­action energy calculation shows that dispersion energy contributes the most to the consolidation of the structure.

## Abstract 2

In the title mol­ecule, the dihedral angles between the central carbonyl­phenyl and adjacent perfluoro­phen­oxy and (tetra­dec­yloxy)benzoate rings are 74.19 (2) and 67.86 (2)°, respectively and the tetra­decyl chain adopts an extended conformation. In the crystal, the mol­ecules are linked by C—H⋯O and C—H⋯F hydrogen bonds, forming *C*(7) and *C*(10) chains, respectively, both running infinitely along [010].

## 1. Chemical context

Benzo­phenone derivatives have been reported to inhibit leukotriene release and have been evaluated as inhibitors of HIV reverse transcriptase, where their activity has been attributed to hydrogen-bonding and π–π inter­actions (Mahendra *et al.*, 2005 ▸). In addition to their pharmaceutical importance, aromatic ester systems such as phenyl benzoates have been widely studied in the field of thermotropic liquid crystals. These materials consist of rigid aromatic cores linked to flexible terminal chains, which have played an important role in governing mesophase formation and stability. Structural modifications have significantly influenced phase behaviour; rigid lateral substituents have tended to disrupt mol­ecular packing, whereas flexible alkyl or alk­oxy chains have modulated phase transitions depending on chain length (Yao *et al.*, 2021 ▸). Furthermore, fluorine substitution has been recognized as an effective strategy for tuning mol­ecular properties, as it can modify dipole moments, enhance thermal and chemical stability, and influence inter­molecular inter­actions.

Recently, liquid-crystalline materials have gained increasing attention due to their inter­actions with biological systems. Studies have indicated that such materials have influenced biological activity by reducing bacterial viability and affecting biochemical pathways such as peroxisome proliferator-activated receptor gamma (PPARγ) regulation (Li *et al.*, 2024 ▸). Finally, alkyl chains have played a significant role in enhancing the biological performance of organic mol­ecules by improving cell membrane permeability. Increased chain length has been associated with improved anti­cancer, anti-tuberculosis, and anti-inflammatory activities, owing to better inter­action with biological targets (Devarajegowda *et al.*, 2025 ▸). As part of our studies in this area, we now describe the synthesis and structure of the title compound, C_34_H_37_F_5_O_5_ (**I**).
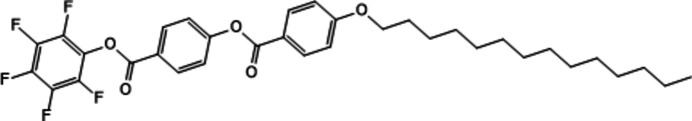


## 2. Structural commentary

**Figure 1:**
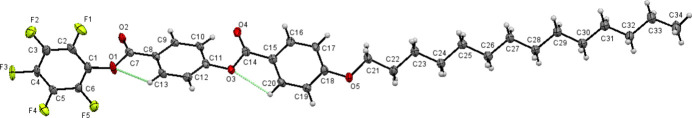
The mol­ecular structure of (**I**) showing 50% probability ellipsoids.

The mol­ecular structure of (**I**) is presented in Fig. 1 ▸. The dihedral angle between the perfluoro­phen­oxy ring (atoms C1–C6) and central carbonyl­phenyl (C8–C13) and (tetra­dec­yloxy)benzoate (C15–C20) rings are 74.19 (2) and 67.86 (2)°, respectively, indicating that the central aromatic ring is approximately normal to the two adjacent rings. The dihedral angle between the outer rings of 6.93 (3)° indicates that they are approximately parallel to each other. The torsion angle associated with the ester groups between the perfluoro­phen­oxy and carbonyl­phenyl, and carbonyl­phenyl and (tetra­dec­yloxy)benzoate rings are −175.8 (3)° for C8—C7—O1—C1 and −172.5 (3)° for C15—C14—O3—C11, whereas the C18—O5—C21—C22 torsion angle across the oxygen atom of the (tetra­dec­yloxy)benzoate ring and the C_14_ alkyl chain is found to be 179.9 (3)°. Otherwise the bond lengths and angles are normal. Two short intra­molecular C—H⋯O contacts (Table 1 ▸) may help to consolidate the mol­ecular conformation.

## 3. Supra­molecular features

**Figure 2:**
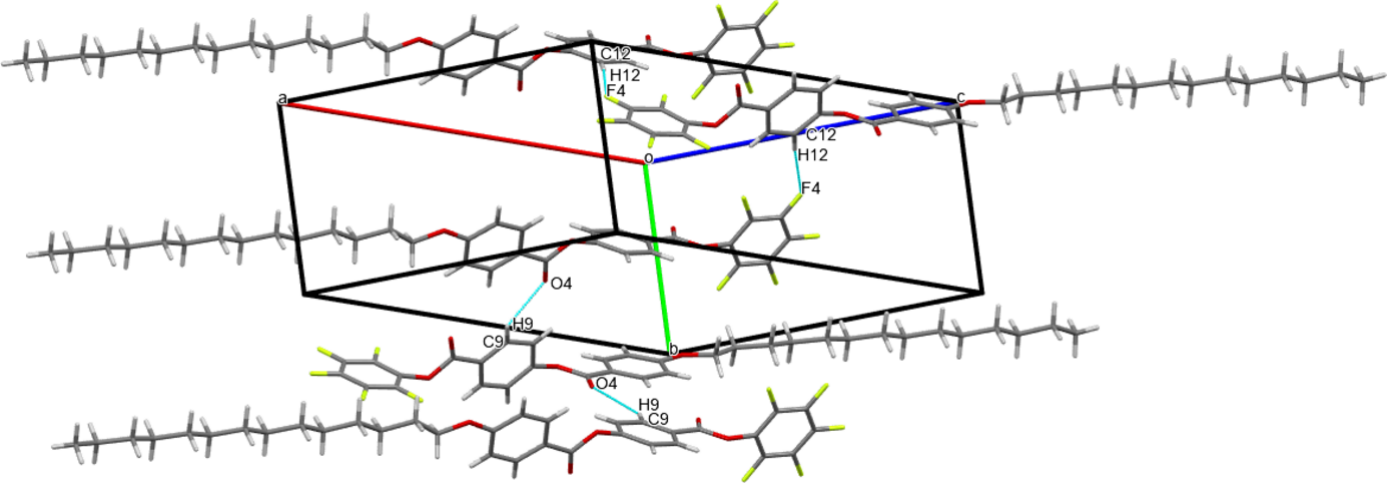
The packing diagram of (**I**) showing C—H⋯O and C—H⋯F hydrogen bonds as blue dashed lines.

In the extended structure of (**I**), a C9—H9⋯O4 hydrogen bond (Table 1 ▸) connects mol­ecules into a *C*(7) chain propagating along [010]. The chain is consolidated by a C12—H12⋯F4 hydrogen bond, which generates a C(10) chain (Fig. 2 ▸). Three C—F⋯π inter­actions, namely C3—F2⋯*Cg*3, C5—F4⋯*Cg*3 and C6—F5⋯*Cg*2 where *Cg*2 and *Cg*3 are the centroids of central carbonyl­phenyl and (tetra­dec­yloxy)benzoate rings, respectively, are seen. Weak aromatic π–π stacking inter­actions, namely *Cg*1⋯*Cg*3, with a centroid–centroid distance of 4.078 (3) Å (slippage = 2.583 Å) and *Cg*2⋯*Cg*2 [centroid–centroid separation = 3.792 (3) Å, slippage = 1.726 Å], where *Cg*1 is centroid of the perfluoro­phen­oxy ring (see supplementary figures) may help to consolidate the packing.

## 4. Database survey

A search of the Cambridge Structural Database (CSD, version 6.01, March 2026; Groom *et al.*, 2016 ▸) for structures containing the phenyl benzoate moiety yielded more than 30 hits. Among these, five closely related structures with CSD refcodes HEKLAN (Dey *et al.*, 2017 ▸), MEXCOJ (Ambekar *et al.*, 2013 ▸), OQALOL (Mandal *et al.*, 2025 ▸), CIKTEW (Gowda *et al.*, 2007 ▸), and KUTGOW (Moumou *et al.*, 2010 ▸) feature substituted aromatic rings or long alkyl chains. In these structures, the dihedral angles between the phenyl ring and the aromatic ring of the benzoate moiety lie between 62 and 76° compared to 67.86 (2)° in (**I**). In all these structures, the ester linkages adopt their expected conformations with C—C—O—C torsion angles close to 180°.

## 5. Hirshfeld surface analysis

**Figure 3:**
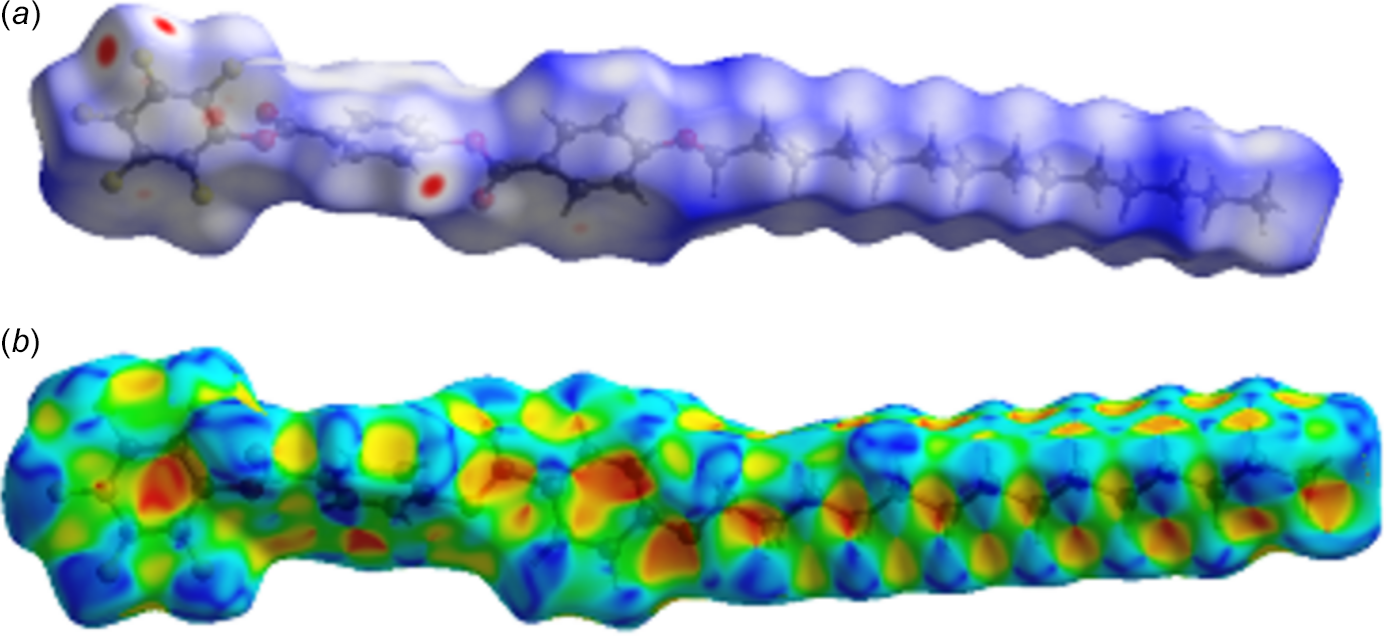
Views of the three-dimensional Hirshfeld surface of (**I**) mapped over (*a*) *d*_norm_ and (*b*) shape-index.

**Figure 4:**
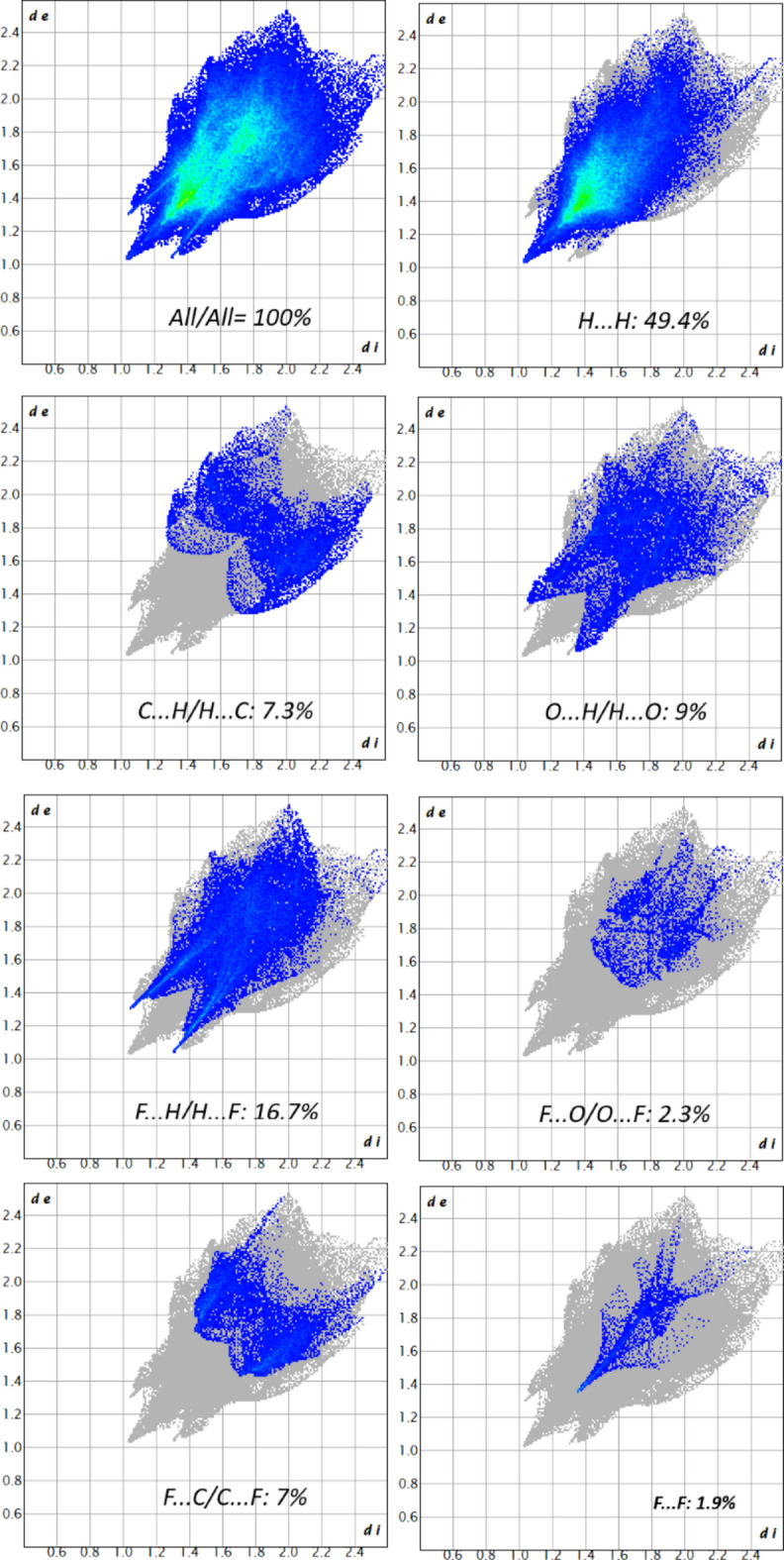
The two-dimensional fingerprint plots for (**I**), showing the contributions of the different contact types to the Hirshfeld surface.

**Figure 5:**
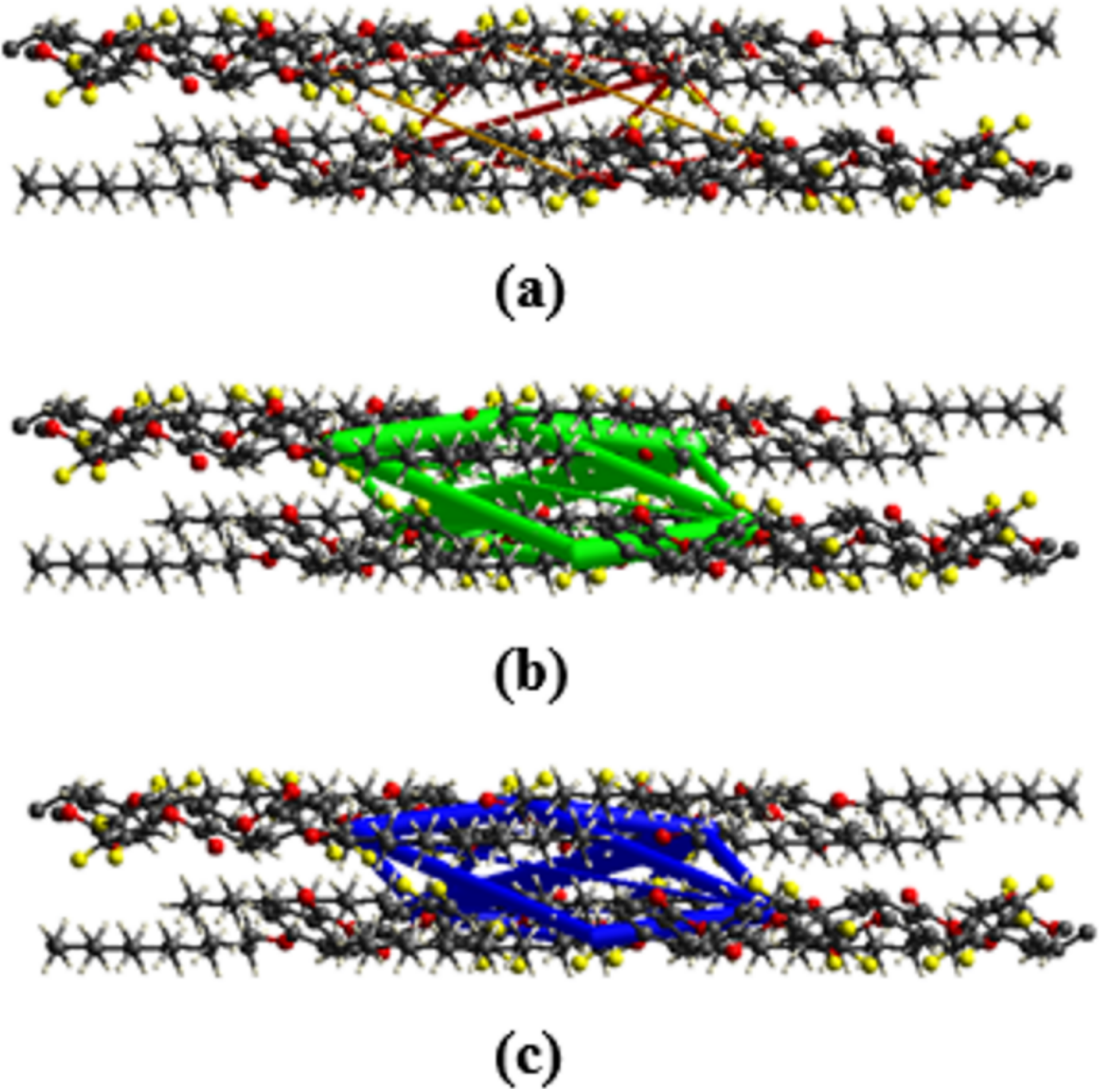
The energy frameworks for the inter­action energies of (**I**): (*a*) Coloumbic energy, (*b*) dispersion energy and (*c*) total energy.

The Hirshfeld surface analysis of (**I**), mapped over *d*_norm_, obtained using *CrystalExplorer* (Spackman *et al.*, 2021 ▸), is presented in Fig. 3 ▸. The two-dimensional fingerprint plots indicate that the contributions to the crystal packing are from H⋯H: (49.4%), F⋯H/H⋯F: (16.7%), C⋯H/H⋯C: (7.3%), C⋯F/F⋯C: (7%) F⋯O/O⋯F: (2.3%), F⋯F: (1.9%) contacts as shown in Fig. 4 ▸. The inter­action energies were computed for (**I**) using the basis set B3LYP\631-G(d,p) for the mol­ecular pairs within a cluster of 3.8 Å radius. The net inter­action energies were calculated as *E_ele_ =* −59.6 kJ mol^−1^, *E_pol_ =* −14.1 kJ mol^−1^, *E_dis_ =* −464.8 kJ mol^−1^, *E_rep_ = +*142.2 kJ mol^−1^ and total inter­action energy *E_tot_ = -*390.4 kJ mol^−1^. The overall inter­action energy is strongly negative, confirming that the crystal packing is energetically favourable and primarily governed by dispersion forces. The topology of energy frameworks for the Coloumbic, dispersion and total energies are shown in Fig. 5 ▸.

## 6. Synthesis and crystallization

A reaction mixture of 2,3,4,5,6-penta­fluoro­phenol (0.184 g, 1 eq) and 4-{[4-(tetra­dec­yloxy)benzo­yl]­oxy}benzoic acid (0.454 g, 1 eq) in di­chloro­methane was stirred at room temperature overnight using the DCC esterification process in the presence of *N*,*N*-di­methyl­amino­pyrimidine as a catalyst. The insoluble byproduct di­cyclo­hexyl urea was removed by filtration. The filtrate was washed with 5% acetic acid solution in water and then with pure water. The filtrate was passed through silica gel, and then left undisturbed for a week to grow crystals of (**I**) for X-ray studies. ^1^H NMR (500 MHz, CDCl_3_): δ 8.12–8.02 (*m*, 4H, Ar-H), 7.54 (*m*, 2H, Ar-H), 7.10 (*d*, *J* = 8.5Hz, 2H, Ar-H), 4.01 (*t*, *J* = 6.5Hz, 2H, –OCH_2_–), 1.74–1.25 (*m*, 24H, –CH_2_–alk­yl), 0.91 (*t*, *J* = 4.5Hz, 3H, –CH_3_) ppm. Elemental analysis (%) calculated: C 65.80; H 6.01; F 15.31; O 12.89; found C 65.85; H 6.05; F 15.28%. Since the title compound has liquid crystal properties, results will be reported in due course.

## 7. Refinement

Crystal data, data collection and structure refinement details are summarized in Table 2 ▸. The hydrogen-atom positions were calculated geometrically (C—H = 0.93–0.97 Å) and refined using a riding model by applying the constraint *U*_iso_(H) = 1.2*U*_eq_(C) or 1.5*U*_eq_(methyl C).

## Supplementary Material

Crystal structure: contains datablock(s) I. DOI: 10.1107/S2056989026006213/hb8225sup1.cif

Structure factors: contains datablock(s) I. DOI: 10.1107/S2056989026006213/hb8225Isup2.hkl

Supplementary packing figures. DOI: 10.1107/S2056989026006213/hb8225sup3.docx

Supporting information file. DOI: 10.1107/S2056989026006213/hb8225Isup4.cml

CCDC reference: 2561963

Additional supporting information:  crystallographic information; 3D view; checkCIF report

## Figures and Tables

**Table 1:** Hydrogen-bond geometry (Å, °) *Cg*1, *Cg*2 and *Cg*3 are the centroids of the C1–C6, C8–C13 and C15–C20 rings, respectively.

*D*—H⋯*A*	*D*—H	H⋯*A*	*D*⋯*A*	*D*—H⋯*A*
C20—H20⋯O3	0.93	2.45	2.753 (4)	99
C13—H13⋯O1	0.93	2.40	2.720 (4)	100
C9—H9⋯O4^i^	0.93	2.52	3.177 (4)	128
C12—H12⋯F4^ii^	0.93	2.50	3.425 (4)	172
C3—F2⋯*Cg*3^iii^	1.33 (1)	3.30 (1)	3.634 (4)	94 (1)
C5—F4⋯*Cg*3^iv^	1.34 (1)	3.15 (1)	3.385 (4)	88 (1)
C6—F5⋯*Cg*2^v^	1.35 (1)	3.48 (1)	4.077 (4)	107 (1)

Symmetry codes: (i) 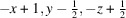; (ii) 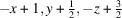; (iii) 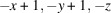; (iv) 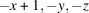; (v) 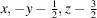.

**Table 2:** Experimental details

Crystal data	
Chemical formula	C_34_H_37_F_5_O_5_
*M*_r_	620.66
Crystal system, space group	Monoclinic, *P*2_1_/*c*
Temperature (K)	423
*a*, *b*, *c* (Å)	27.811 (16), 8.206 (5), 13.975 (8)
β (°)	103.868 (14)
*V* (Å^3^)	3096 (3)
*Z*	4
Radiation type	Mo *K*α
μ (mm^−1^)	0.11
Crystal size (mm)	0.43 × 0.32 × 0.27

Data collection	
Diffractometer	Bruker *SMART* APEXII CCD
Absorption correction	Multi-scan (*SADABS*; Krause *et al.*, 2015 ▸)
*T*_min_, *T*_max_	0.954, 0.970
No. of measured, independent and observed [*I* > 2σ(*I*)] reflections	28865, 5466, 4231
*R*_int_	0.063
(sin θ/λ)_max_ (Å^−1^)	0.595

Refinement	
*R*[*F*^2^ > 2σ(*F*^2^)], *wR*(*F*^2^), *S*	0.078, 0.165, 1.15
No. of reflections	5466
No. of parameters	398
H-atom treatment	H-atom parameters constrained
Δρ_max_, Δρ_min_ (e Å^−3^)	0.25, −0.29

Computer programs: *APEX2* and *SAINT* (Bruker, 2017 ▸), *SHELXT2018/3* (Sheldrick, 2015*a* ▸), *SHELXL2019/2* (Sheldrick, 2015*b* ▸), *Mercury* (Macrae *et al.*, 2020 ▸) and *publCIF* (Westrip,2010 ▸).

## supplementary crystallographic information

### 4-[(2,3,4,5,6-Pentafluorophenoxy)carbonyl]phenyl 4-(tetradecyloxy)benzoate Crystal data

**Table:**

C_34_H_37_F_5_O_5_	prism
*M**_r_* = 620.66	*D*_x_ = 1.331 Mg m^−^^3^
Monoclinic, *P*2_1_/*c*	Mo *K*α radiation, λ = 0.71073 Å
*a* = 27.811 (16) Å	Cell parameters from 4231 reflections
*b* = 8.206 (5) Å	θ = 2.5–25.0°
*c* = 13.975 (8) Å	µ = 0.11 mm^−^^1^
β = 103.868 (14)°	*T* = 423 K
*V* = 3096 (3) Å^3^	Prism, colourless
*Z* = 4	0.43 × 0.32 × 0.27 mm
*F*(000) = 1304	

### 4-[(2,3,4,5,6-Pentafluorophenoxy)carbonyl]phenyl 4-(tetradecyloxy)benzoate Data collection

**Table:**

Bruker SMART APEXII CCD diffractometer	5466 independent reflections
Radiation source: fine-focus sealed tube	4231 reflections with *I* > 2σ(*I*)
Graphite monochromator	*R*_int_ = 0.063
Detector resolution: 2.09 pixels mm^-1^	θ_max_ = 25.0°, θ_min_ = 2.6°
φ and Ω scans	*h* = −32→33
Absorption correction: multi-scan (SADABS; Krause *et al.*, 2015)	*k* = −9→9
*T*_min_ = 0.954, *T*_max_ = 0.970	*l* = −15→16
28865 measured reflections	

### 4-[(2,3,4,5,6-Pentafluorophenoxy)carbonyl]phenyl 4-(tetradecyloxy)benzoate Refinement

**Table:**

Refinement on *F*^2^	Secondary atom site location: difference Fourier map
Least-squares matrix: full	Hydrogen site location: inferred from neighbouring sites
*R*[*F*^2^ > 2σ(*F*^2^)] = 0.078	H-atom parameters constrained
*wR*(*F*^2^) = 0.165	*w* = 1/[σ^2^(*F*_o_^2^) + (0.0504*P*)^2^ + 4.4152*P*] where *P* = (*F*_o_^2^ + 2*F*_c_^2^)/3
*S* = 1.15	(Δ/σ)_max_ < 0.001
5466 reflections	Δρ_max_ = 0.25 e Å^−^^3^
398 parameters	Δρ_min_ = −0.29 e Å^−^^3^
0 restraints	Extinction correction: SHELXL2019/2 (Sheldrick 2015b), Fc^*^=kFc[1+0.001xFc^2^λ^3^/sin(2θ)]^-1/4^
0 constraints	Extinction coefficient: 0.0020 (6)
Primary atom site location: structure-invariant direct methods	

### 4-[(2,3,4,5,6-Pentafluorophenoxy)carbonyl]phenyl 4-(tetradecyloxy)benzoate Special details

**Table:**

Geometry. All esds (except the esd in the dihedral angle between two l.s. planes) are estimated using the full covariance matrix. The cell esds are taken into account individually in the estimation of esds in distances, angles and torsion angles; correlations between esds in cell parameters are only used when they are defined by crystal symmetry. An approximate (isotropic) treatment of cell esds is used for estimating esds involving l.s. planes.

### 4-[(2,3,4,5,6-Pentafluorophenoxy)carbonyl]phenyl 4-(tetradecyloxy)benzoate Fractional atomic coordinates and isotropic or equivalent isotropic displacement parameters (Å^2^)

**Table:**

	*x*	*y*	*z*	*U*_iso_*/*U*_eq_	
O2	0.38024 (8)	0.6170 (3)	0.48615 (16)	0.0262 (5)	
F4	0.35716 (8)	0.5541 (2)	0.87454 (14)	0.0381 (5)	
F5	0.42775 (7)	0.5653 (2)	0.77188 (14)	0.0353 (5)	
O3	0.59440 (7)	0.7225 (3)	0.40331 (16)	0.0251 (5)	
O1	0.41978 (8)	0.7700 (3)	0.61509 (17)	0.0345 (6)	
O5	0.77655 (8)	0.6657 (3)	0.21290 (16)	0.0300 (6)	
C8	0.46276 (11)	0.7110 (3)	0.4931 (2)	0.0183 (7)	
C1	0.38045 (12)	0.7534 (4)	0.6592 (2)	0.0266 (8)	
C27	0.96003 (12)	0.6387 (5)	−0.2196 (2)	0.0310 (8)	
H27A	0.944781	0.736989	−0.251815	0.037*	
H27B	0.943268	0.546417	−0.256432	0.037*	
C14	0.59713 (12)	0.7989 (4)	0.3177 (2)	0.0230 (7)	
C15	0.64528 (11)	0.7663 (4)	0.2936 (2)	0.0219 (7)	
C26	0.95197 (12)	0.6331 (5)	−0.1166 (2)	0.0317 (8)	
H26A	0.970501	0.721440	−0.078670	0.038*	
H26B	0.965523	0.531679	−0.085900	0.038*	
F2	0.25856 (8)	0.9160 (3)	0.64890 (16)	0.0486 (6)	
C28	1.01423 (12)	0.6359 (5)	−0.2243 (2)	0.0327 (8)	
H28A	1.030786	0.729941	−0.189119	0.039*	
H28B	1.029729	0.539202	−0.190492	0.039*	
F1	0.33047 (9)	0.9358 (3)	0.54875 (15)	0.0470 (6)	
C7	0.41639 (12)	0.6913 (4)	0.5266 (2)	0.0208 (7)	
C22	0.83173 (12)	0.6435 (4)	0.1067 (2)	0.0296 (8)	
H22A	0.837076	0.530673	0.127156	0.036*	
H22B	0.856248	0.709328	0.151254	0.036*	
O4	0.56406 (8)	0.8788 (3)	0.27024 (17)	0.0318 (6)	
C11	0.54874 (11)	0.7260 (4)	0.4293 (2)	0.0208 (7)	
C31	1.08395 (12)	0.6394 (5)	−0.4360 (3)	0.0327 (8)	
H31A	1.070576	0.537908	−0.466831	0.039*	
H31B	1.065120	0.727644	−0.473462	0.039*	
C10	0.50888 (11)	0.6382 (4)	0.3745 (2)	0.0211 (7)	
H10	0.511179	0.585478	0.316830	0.025*	
C24	0.89144 (12)	0.6334 (4)	−0.0056 (2)	0.0287 (8)	
H24A	0.901917	0.526046	0.020164	0.034*	
H24B	0.912800	0.712690	0.035420	0.034*	
C32	1.13746 (12)	0.6541 (5)	−0.4426 (3)	0.0315 (8)	
H32A	1.150637	0.757311	−0.414001	0.038*	
H32B	1.156626	0.567831	−0.403756	0.038*	
C23	0.83867 (12)	0.6616 (4)	0.0023 (2)	0.0290 (8)	
H23A	0.817124	0.584640	−0.040248	0.035*	
H23B	0.828538	0.770368	−0.021307	0.035*	
F3	0.27248 (8)	0.7280 (3)	0.81361 (16)	0.0456 (6)	
C20	0.68342 (11)	0.6751 (4)	0.3539 (2)	0.0238 (7)	
H20	0.679478	0.633695	0.413501	0.029*	
C13	0.50303 (12)	0.7993 (4)	0.5465 (2)	0.0242 (7)	
H13	0.500851	0.853027	0.603950	0.029*	
C30	1.07635 (12)	0.6437 (5)	−0.3320 (2)	0.0322 (8)	
H30A	1.090985	0.742796	−0.299860	0.039*	
H30B	1.093700	0.552203	−0.295247	0.039*	
C9	0.46602 (11)	0.6307 (4)	0.4067 (2)	0.0193 (7)	
H9	0.439060	0.571916	0.370932	0.023*	
C19	0.72662 (12)	0.6467 (4)	0.3252 (2)	0.0256 (7)	
H19	0.752020	0.588183	0.366316	0.031*	
C5	0.35055 (12)	0.6489 (4)	0.7945 (2)	0.0247 (7)	
C16	0.65249 (12)	0.8267 (4)	0.2043 (2)	0.0253 (7)	
H16	0.627599	0.888183	0.163886	0.030*	
C6	0.38649 (11)	0.6553 (4)	0.7414 (2)	0.0227 (7)	
C17	0.69537 (12)	0.7975 (4)	0.1749 (2)	0.0260 (8)	
H17	0.699454	0.839085	0.115490	0.031*	
C25	0.89840 (12)	0.6459 (4)	−0.1102 (2)	0.0292 (8)	
H25A	0.879485	0.559996	−0.149846	0.035*	
H25B	0.885100	0.749354	−0.138261	0.035*	
C18	0.73277 (11)	0.7049 (4)	0.2349 (2)	0.0235 (7)	
C12	0.54617 (12)	0.8074 (4)	0.5145 (2)	0.0240 (7)	
H12	0.573131	0.866797	0.549667	0.029*	
C33	1.14389 (13)	0.6441 (5)	−0.5472 (3)	0.0375 (9)	
H33A	1.123245	0.726665	−0.586683	0.045*	
H33B	1.132153	0.538652	−0.574443	0.045*	
C29	1.02213 (12)	0.6368 (5)	−0.3279 (3)	0.0313 (8)	
H29A	1.005045	0.730135	−0.362958	0.038*	
H29B	1.007275	0.539454	−0.361860	0.038*	
C3	0.30040 (12)	0.8311 (4)	0.6789 (3)	0.0286 (8)	
C21	0.78072 (12)	0.6952 (4)	0.1137 (2)	0.0294 (8)	
H21A	0.775738	0.809945	0.097782	0.035*	
H21B	0.755791	0.633271	0.067471	0.035*	
C34	1.19685 (14)	0.6671 (5)	−0.5561 (3)	0.0488 (11)	
H34A	1.197913	0.659169	−0.624146	0.073*	
H34B	1.208594	0.772407	−0.531093	0.073*	
H34C	1.217514	0.584132	−0.518835	0.073*	
C4	0.30756 (12)	0.7354 (4)	0.7631 (3)	0.0286 (8)	
C2	0.33696 (13)	0.8384 (4)	0.6280 (2)	0.0299 (8)	

### 4-[(2,3,4,5,6-Pentafluorophenoxy)carbonyl]phenyl 4-(tetradecyloxy)benzoate Atomic displacement parameters (Å^2^)

**Table:**

	*U*^11^	*U*^22^	*U*^33^	*U*^12^	*U*^13^	*U*^23^
O2	0.0286 (12)	0.0312 (13)	0.0225 (12)	−0.0024 (11)	0.0136 (10)	−0.0085 (10)
F4	0.0579 (14)	0.0315 (11)	0.0309 (12)	0.0028 (10)	0.0222 (10)	0.0050 (9)
F5	0.0306 (11)	0.0402 (12)	0.0363 (12)	0.0074 (9)	0.0104 (9)	−0.0101 (10)
O3	0.0229 (12)	0.0322 (13)	0.0238 (12)	0.0042 (10)	0.0126 (9)	0.0100 (10)
O1	0.0363 (14)	0.0457 (15)	0.0301 (14)	−0.0156 (12)	0.0246 (11)	−0.0174 (12)
O5	0.0225 (12)	0.0443 (15)	0.0270 (13)	0.0043 (11)	0.0135 (10)	0.0052 (11)
C8	0.0273 (17)	0.0141 (14)	0.0153 (16)	0.0005 (13)	0.0084 (13)	0.0006 (12)
C1	0.0275 (18)	0.0291 (18)	0.0272 (18)	−0.0072 (15)	0.0144 (14)	−0.0115 (15)
C27	0.0261 (18)	0.044 (2)	0.0242 (19)	−0.0005 (16)	0.0094 (14)	0.0019 (16)
C14	0.0278 (18)	0.0184 (16)	0.0253 (18)	−0.0006 (14)	0.0113 (14)	0.0009 (14)
C15	0.0261 (17)	0.0170 (16)	0.0243 (17)	−0.0011 (13)	0.0092 (14)	−0.0010 (13)
C26	0.0285 (18)	0.042 (2)	0.0262 (19)	0.0014 (16)	0.0100 (15)	0.0040 (17)
F2	0.0381 (12)	0.0579 (15)	0.0480 (14)	0.0220 (11)	0.0069 (10)	−0.0046 (12)
C28	0.0263 (18)	0.048 (2)	0.0249 (19)	0.0040 (17)	0.0089 (14)	0.0027 (17)
F1	0.0722 (16)	0.0470 (13)	0.0250 (12)	0.0064 (12)	0.0180 (11)	0.0074 (10)
C7	0.0307 (18)	0.0146 (15)	0.0179 (17)	0.0019 (14)	0.0075 (14)	−0.0003 (13)
C22	0.0228 (17)	0.037 (2)	0.031 (2)	−0.0023 (15)	0.0109 (14)	−0.0016 (16)
O4	0.0288 (13)	0.0400 (14)	0.0319 (14)	0.0137 (11)	0.0177 (11)	0.0196 (12)
C11	0.0248 (17)	0.0193 (16)	0.0214 (17)	0.0010 (13)	0.0116 (13)	0.0069 (13)
C31	0.0307 (19)	0.039 (2)	0.030 (2)	0.0021 (16)	0.0104 (15)	0.0015 (16)
C10	0.0299 (18)	0.0195 (16)	0.0163 (16)	0.0019 (14)	0.0104 (13)	−0.0012 (13)
C24	0.0261 (18)	0.038 (2)	0.0246 (18)	0.0018 (16)	0.0108 (14)	0.0027 (16)
C32	0.0298 (19)	0.036 (2)	0.032 (2)	0.0052 (16)	0.0140 (15)	0.0034 (16)
C23	0.0267 (18)	0.037 (2)	0.0262 (19)	0.0008 (15)	0.0117 (14)	0.0000 (15)
F3	0.0407 (12)	0.0570 (14)	0.0526 (14)	0.0028 (11)	0.0374 (11)	−0.0035 (12)
C20	0.0280 (18)	0.0277 (18)	0.0178 (17)	−0.0062 (14)	0.0099 (13)	0.0004 (14)
C13	0.0345 (19)	0.0227 (17)	0.0176 (17)	−0.0008 (15)	0.0102 (14)	−0.0067 (14)
C30	0.0280 (19)	0.043 (2)	0.0273 (19)	0.0022 (16)	0.0102 (15)	0.0027 (16)
C9	0.0237 (16)	0.0189 (15)	0.0158 (16)	−0.0002 (13)	0.0062 (13)	0.0009 (13)
C19	0.0236 (17)	0.0317 (18)	0.0213 (18)	0.0013 (15)	0.0049 (13)	0.0029 (15)
C5	0.0344 (19)	0.0229 (16)	0.0191 (17)	0.0003 (15)	0.0112 (14)	−0.0027 (14)
C16	0.0273 (18)	0.0223 (17)	0.0288 (19)	0.0037 (14)	0.0117 (14)	0.0061 (14)
C6	0.0212 (16)	0.0232 (17)	0.0246 (18)	0.0013 (14)	0.0073 (13)	−0.0082 (14)
C17	0.0281 (18)	0.0315 (18)	0.0218 (18)	0.0022 (15)	0.0124 (14)	0.0080 (15)
C25	0.0267 (18)	0.0355 (19)	0.0261 (19)	0.0022 (15)	0.0074 (14)	0.0019 (15)
C18	0.0224 (17)	0.0238 (16)	0.0267 (18)	−0.0066 (14)	0.0107 (14)	−0.0033 (14)
C12	0.0245 (17)	0.0225 (17)	0.0245 (18)	−0.0042 (14)	0.0050 (14)	0.0002 (14)
C33	0.038 (2)	0.046 (2)	0.033 (2)	0.0020 (18)	0.0182 (17)	0.0009 (18)
C29	0.0274 (18)	0.040 (2)	0.0293 (19)	0.0017 (16)	0.0119 (15)	0.0022 (16)
C3	0.0291 (18)	0.0298 (19)	0.0272 (19)	0.0047 (15)	0.0074 (15)	−0.0086 (15)
C21	0.0302 (19)	0.036 (2)	0.0258 (19)	0.0019 (16)	0.0138 (15)	0.0021 (16)
C34	0.047 (2)	0.057 (3)	0.053 (3)	0.004 (2)	0.032 (2)	0.007 (2)
C4	0.0271 (18)	0.0342 (19)	0.031 (2)	−0.0054 (15)	0.0191 (15)	−0.0130 (16)
C2	0.044 (2)	0.0300 (19)	0.0180 (18)	−0.0052 (16)	0.0122 (15)	−0.0046 (15)

### 4-[(2,3,4,5,6-Pentafluorophenoxy)carbonyl]phenyl 4-(tetradecyloxy)benzoate Geometric parameters (Å, º)

**Table:**

O2—C7	1.196 (4)	C24—C23	1.516 (4)
F4—C5	1.339 (4)	C24—C25	1.524 (4)
F5—C6	1.345 (4)	C24—H24A	0.9700
O3—C14	1.369 (4)	C24—H24B	0.9700
O3—C11	1.403 (4)	C32—C33	1.517 (5)
O1—C7	1.378 (4)	C32—H32A	0.9700
O1—C1	1.385 (4)	C32—H32B	0.9700
O5—C18	1.363 (4)	C23—H23A	0.9700
O5—C21	1.439 (4)	C23—H23B	0.9700
C8—C13	1.391 (4)	F3—C4	1.335 (4)
C8—C9	1.397 (4)	C20—C19	1.374 (4)
C8—C7	1.482 (4)	C20—H20	0.9300
C1—C2	1.374 (5)	C13—C12	1.379 (4)
C1—C6	1.379 (5)	C13—H13	0.9300
C27—C26	1.510 (4)	C30—C29	1.524 (4)
C27—C28	1.525 (4)	C30—H30A	0.9700
C27—H27A	0.9700	C30—H30B	0.9700
C27—H27B	0.9700	C9—H9	0.9300
C14—O4	1.193 (4)	C19—C18	1.398 (4)
C14—C15	1.481 (4)	C19—H19	0.9300
C15—C16	1.402 (4)	C5—C4	1.369 (5)
C15—C20	1.403 (4)	C5—C6	1.381 (4)
C26—C25	1.517 (4)	C16—C17	1.372 (4)
C26—H26A	0.9700	C16—H16	0.9300
C26—H26B	0.9700	C17—C18	1.394 (5)
F2—C3	1.335 (4)	C17—H17	0.9300
C28—C29	1.515 (5)	C25—H25A	0.9700
C28—H28A	0.9700	C25—H25B	0.9700
C28—H28B	0.9700	C12—H12	0.9300
F1—C2	1.343 (4)	C33—C34	1.520 (5)
C22—C21	1.506 (4)	C33—H33A	0.9700
C22—C23	1.523 (5)	C33—H33B	0.9700
C22—H22A	0.9700	C29—H29A	0.9700
C22—H22B	0.9700	C29—H29B	0.9700
C11—C12	1.381 (4)	C3—C2	1.375 (5)
C11—C10	1.388 (4)	C3—C4	1.389 (5)
C31—C32	1.518 (5)	C21—H21A	0.9700
C31—C30	1.519 (5)	C21—H21B	0.9700
C31—H31A	0.9700	C34—H34A	0.9600
C31—H31B	0.9700	C34—H34B	0.9600
C10—C9	1.373 (4)	C34—H34C	0.9600
C10—H10	0.9300		
			
C14—O3—C11	117.4 (2)	C22—C23—H23B	108.8
C7—O1—C1	117.5 (3)	H23A—C23—H23B	107.7
C18—O5—C21	117.4 (2)	C19—C20—C15	120.2 (3)
C13—C8—C9	119.9 (3)	C19—C20—H20	119.9
C13—C8—C7	122.5 (3)	C15—C20—H20	119.9
C9—C8—C7	117.6 (3)	C12—C13—C8	120.1 (3)
C2—C1—C6	118.9 (3)	C12—C13—H13	119.9
C2—C1—O1	122.5 (3)	C8—C13—H13	119.9
C6—C1—O1	118.5 (3)	C31—C30—C29	113.6 (3)
C2—C1—O1	122.5 (3)	C31—C30—H30A	108.8
C6—C1—O1	118.5 (3)	C29—C30—H30A	108.8
C26—C27—C28	114.5 (3)	C31—C30—H30B	108.8
C26—C27—H27A	108.6	C29—C30—H30B	108.8
C28—C27—H27A	108.6	H30A—C30—H30B	107.7
C26—C27—H27B	108.6	C10—C9—C8	120.2 (3)
C28—C27—H27B	108.6	C10—C9—H9	119.9
H27A—C27—H27B	107.6	C8—C9—H9	119.9
O4—C14—O3	122.7 (3)	C20—C19—C18	120.7 (3)
O4—C14—O3	122.7 (3)	C20—C19—H19	119.7
O4—C14—C15	126.3 (3)	C18—C19—H19	119.7
O3—C14—C15	111.0 (3)	F4—C5—C4	120.2 (3)
O3—C14—C15	111.0 (3)	F4—C5—C6	119.9 (3)
C16—C15—C20	118.4 (3)	C4—C5—C6	119.8 (3)
C16—C15—C14	117.9 (3)	C17—C16—C15	121.6 (3)
C20—C15—C14	123.7 (3)	C17—C16—H16	119.2
C27—C26—C25	115.2 (3)	C15—C16—H16	119.2
C27—C26—H26A	108.5	F5—C6—C1	120.6 (3)
C25—C26—H26A	108.5	F5—C6—C5	118.8 (3)
C27—C26—H26B	108.5	C1—C6—C5	120.7 (3)
C25—C26—H26B	108.5	C16—C17—C18	119.5 (3)
H26A—C26—H26B	107.5	C16—C17—H17	120.2
C29—C28—C27	114.3 (3)	C18—C17—H17	120.2
C29—C28—H28A	108.7	C26—C25—C24	113.8 (3)
C27—C28—H28A	108.7	C26—C25—H25A	108.8
C29—C28—H28B	108.7	C24—C25—H25A	108.8
C27—C28—H28B	108.7	C26—C25—H25B	108.8
H28A—C28—H28B	107.6	C24—C25—H25B	108.8
O2—C7—O1	122.1 (3)	H25A—C25—H25B	107.7
O2—C7—O1	122.1 (3)	O5—C18—C17	124.8 (3)
O2—C7—C8	127.1 (3)	O5—C18—C19	115.6 (3)
O1—C7—C8	110.8 (3)	C17—C18—C19	119.6 (3)
O1—C7—C8	110.8 (3)	C13—C12—C11	119.0 (3)
C21—C22—C23	111.8 (3)	C13—C12—H12	120.5
C21—C22—H22A	109.2	C11—C12—H12	120.5
C23—C22—H22A	109.2	C32—C33—C34	114.4 (3)
C21—C22—H22B	109.2	C32—C33—H33A	108.7
C23—C22—H22B	109.2	C34—C33—H33A	108.7
H22A—C22—H22B	107.9	C32—C33—H33B	108.7
C12—C11—C10	121.8 (3)	C34—C33—H33B	108.7
C12—C11—O3	118.0 (3)	H33A—C33—H33B	107.6
C10—C11—O3	119.9 (3)	C28—C29—C30	114.1 (3)
C12—C11—O3	118.0 (3)	C28—C29—H29A	108.7
C10—C11—O3	119.9 (3)	C30—C29—H29A	108.7
C32—C31—C30	114.8 (3)	C28—C29—H29B	108.7
C32—C31—H31A	108.6	C30—C29—H29B	108.7
C30—C31—H31A	108.6	H29A—C29—H29B	107.6
C32—C31—H31B	108.6	F2—C3—C2	120.8 (3)
C30—C31—H31B	108.6	F2—C3—C4	119.9 (3)
H31A—C31—H31B	107.5	C2—C3—C4	119.3 (3)
C9—C10—C11	118.9 (3)	O5—C21—C22	108.1 (3)
C9—C10—H10	120.6	O5—C21—H21A	110.1
C11—C10—H10	120.6	C22—C21—H21A	110.1
C23—C24—C25	114.1 (3)	O5—C21—H21B	110.1
C23—C24—H24A	108.7	C22—C21—H21B	110.1
C25—C24—H24A	108.7	H21A—C21—H21B	108.4
C23—C24—H24B	108.7	C33—C34—H34A	109.5
C25—C24—H24B	108.7	C33—C34—H34B	109.5
H24A—C24—H24B	107.6	H34A—C34—H34B	109.5
C33—C32—C31	113.4 (3)	C33—C34—H34C	109.5
C33—C32—H32A	108.9	H34A—C34—H34C	109.5
C31—C32—H32A	108.9	H34B—C34—H34C	109.5
C33—C32—H32B	108.9	F3—C4—C5	120.0 (3)
C31—C32—H32B	108.9	F3—C4—C3	119.9 (3)
H32A—C32—H32B	107.7	C5—C4—C3	120.1 (3)
C24—C23—C22	113.7 (3)	F1—C2—C1	119.9 (3)
C24—C23—H23A	108.8	F1—C2—C3	118.9 (3)
C22—C23—H23A	108.8	C1—C2—C3	121.2 (3)
C24—C23—H23B	108.8		
			
C7—O1—C1—C2	−77.5 (4)	C2—C1—C6—C5	−3.3 (5)
C7—O1—C1—C6	106.5 (3)	O1—C1—C6—C5	172.8 (3)
C7—O1—C1—O1	0 (100)	O1—C1—C6—C5	172.8 (3)
C11—O3—C14—O4	6.8 (5)	F4—C5—C6—F5	−0.1 (5)
C11—O3—C14—C15	−172.5 (3)	C4—C5—C6—F5	−178.0 (3)
O4—C14—C15—C16	−3.6 (5)	F4—C5—C6—C1	−179.4 (3)
O3—C14—C15—C16	175.6 (3)	C4—C5—C6—C1	2.7 (5)
O3—C14—C15—C16	175.6 (3)	C15—C16—C17—C18	0.3 (5)
O4—C14—C15—C20	178.0 (3)	C27—C26—C25—C24	177.8 (3)
O3—C14—C15—C20	−2.7 (4)	C23—C24—C25—C26	174.7 (3)
O3—C14—C15—C20	−2.7 (4)	C21—O5—C18—C17	−12.3 (5)
C28—C27—C26—C25	176.5 (3)	C21—O5—C18—C19	168.3 (3)
C26—C27—C28—C29	178.4 (3)	C16—C17—C18—O5	178.9 (3)
C1—O1—C7—O2	3.3 (5)	C16—C17—C18—C19	−1.7 (5)
C1—O1—C7—C8	−175.8 (3)	C20—C19—C18—O5	−178.3 (3)
C13—C8—C7—O2	−179.4 (3)	C20—C19—C18—C17	2.3 (5)
C9—C8—C7—O2	−2.3 (5)	C8—C13—C12—C11	−0.4 (5)
C13—C8—C7—O1	−0.4 (4)	C10—C11—C12—C13	0.9 (5)
C9—C8—C7—O1	176.7 (3)	O3—C11—C12—C13	−173.9 (3)
C13—C8—C7—O1	−0.4 (4)	O3—C11—C12—C13	−173.9 (3)
C9—C8—C7—O1	176.7 (3)	C31—C32—C33—C34	177.1 (3)
C14—O3—C11—C12	−115.7 (3)	C27—C28—C29—C30	176.7 (3)
O3—O3—C11—C10	0.00 (11)	C31—C30—C29—C28	178.3 (3)
C14—O3—C11—C10	69.4 (4)	C18—O5—C21—C22	179.9 (3)
C12—C11—C10—C9	−0.9 (5)	C23—C22—C21—O5	176.9 (3)
O3—C11—C10—C9	173.8 (3)	F4—C5—C4—F3	1.4 (5)
O3—C11—C10—C9	173.8 (3)	C6—C5—C4—F3	179.2 (3)
C30—C31—C32—C33	178.2 (3)	F4—C5—C4—C3	−178.8 (3)
C25—C24—C23—C22	178.1 (3)	C6—C5—C4—C3	−0.9 (5)
C21—C22—C23—C24	172.0 (3)	F2—C3—C4—F3	0.2 (5)
C16—C15—C20—C19	0.0 (5)	C2—C3—C4—F3	179.6 (3)
C14—C15—C20—C19	178.4 (3)	F2—C3—C4—C5	−179.6 (3)
C9—C8—C13—C12	−0.1 (5)	C2—C3—C4—C5	−0.3 (5)
C7—C8—C13—C12	176.9 (3)	C6—C1—C2—F1	179.4 (3)
C32—C31—C30—C29	177.3 (3)	O1—C1—C2—F1	3.5 (5)
C11—C10—C9—C8	0.3 (5)	O1—C1—C2—F1	3.5 (5)
C13—C8—C9—C10	0.2 (5)	C6—C1—C2—C3	2.1 (5)
C7—C8—C9—C10	−177.0 (3)	O1—C1—C2—C3	−173.8 (3)
C15—C20—C19—C18	−1.4 (5)	O1—C1—C2—C3	−173.8 (3)
C20—C15—C16—C17	0.6 (5)	F2—C3—C2—F1	1.8 (5)
C14—C15—C16—C17	−177.9 (3)	C4—C3—C2—F1	−177.6 (3)
C2—C1—C6—F5	177.4 (3)	F2—C3—C2—C1	179.0 (3)
O1—C1—C6—F5	−6.5 (4)	C4—C3—C2—C1	−0.4 (5)
O1—C1—C6—F5	−6.5 (4)		

### 4-[(2,3,4,5,6-Pentafluorophenoxy)carbonyl]phenyl 4-(tetradecyloxy)benzoate Hydrogen-bond geometry (Å, º)

*Cg*1, *Cg*2 and *Cg*3 are the centroids of the C1–C6, C8–C13 and C15–C20 rings, respectively.

**Table:**

*D*—H···*A*	*D*—H	H···*A*	*D*···*A*	*D*—H···*A*
C20—H20···O3	0.93	2.45	2.753 (4)	99
C13—H13···O1	0.93	2.40	2.720 (4)	100
C9—H9···O4^i^	0.93	2.52	3.177 (4)	128
C12—H12···F4^ii^	0.93	2.50	3.425 (4)	172
C3—F2···*Cg*3^iii^	1.33 (1)	3.30 (1)	3.634 (4)	94 (1)
C5—F4···*Cg*3^iv^	1.34 (1)	3.15 (1)	3.385 (4)	88 (1)
C6—F5···*Cg*2^v^	1.35 (1)	3.48 (1)	4.077 (4)	107 (1)

Symmetry codes: (i) −*x*+1, *y*−1/2, −*z*+1/2; (ii) −*x*+1, *y*+1/2, −*z*+3/2; (iii) −*x*+1, −*y*+1, −*z*; (iv) −*x*+1, −*y*, −*z*; (v) *x*, −*y*−1/2, *z*−3/2.
