# Supplementary material for: Synthesis and structure of 4-[(2,3,4,5,6-pentafluoro­phen­oxy)carbon­yl]phenyl 4-(tetra­dec­yloxy)benzoate
Source: Acta Crystallogr E Crystallogr Commun. 2026 Jun 23;82(Pt 7):858–61. doi: 10.1107/S2056989026006213 (PMC13330810; doi:10.1107/S2056989026006213)
Supplement: Supplementary file 3 [file e-82-00858-sup3.docx]

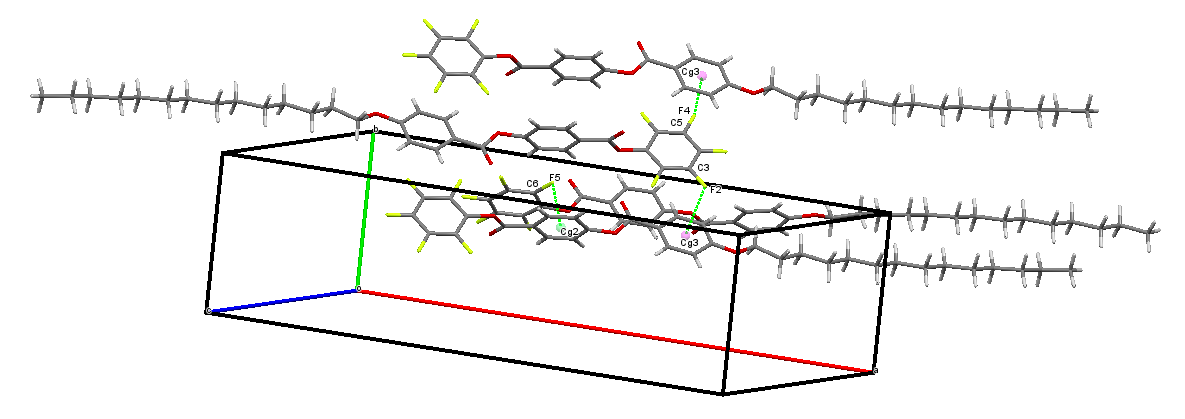


Fig. 6. The Crystal packing of the compound (I), dashed line indicates C—F···π interactions.


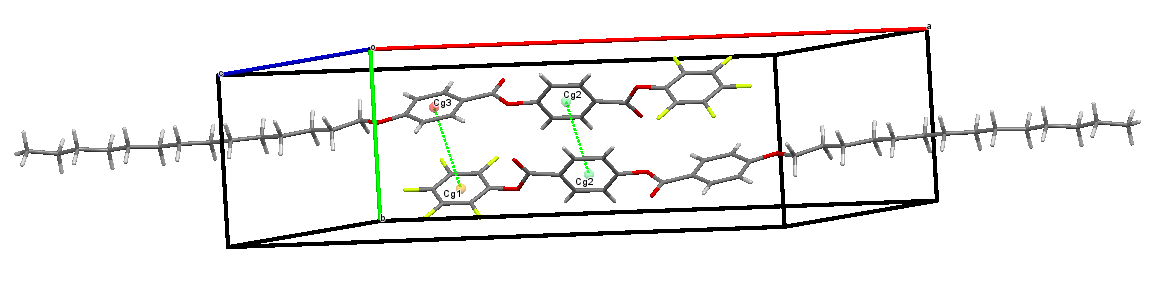


**Fig. 7:** The Crystal packing of the compound (I), with π---π stacking.
